# Strongyloides stercoralis Hyperinfection Presenting As Ascending Cholangitis and Escherichia coli Bacteremia in a Patient With Metastatic Pancreatic Cancer

**DOI:** 10.7759/cureus.90625

**Published:** 2025-08-20

**Authors:** Alan Wang, John Greene

**Affiliations:** 1 Osteopathic Medicine, Nova Southeastern University Dr. Kiran C. Patel College of Osteopathic Medicine, Clearwater, USA; 2 Infectious Diseases, Moffitt Cancer Center, Tampa, USA

**Keywords:** cholangitis, e. coli bacteremia, hyperinfection, strongyloides stercoralis, strongyloidiasis

## Abstract

*Strongyloides stercoralis* is a nematode capable of persisting in humans for years to decades, or even lifelong, through an autoinfection cycle. In immunocompromised individuals, this can lead to uncontrolled proliferation and hyperinfection syndrome. We report the case of a 56-year-old Jamaican man with metastatic pancreatic cancer status post-Whipple procedure who presented with fever, sepsis, and signs of ascending cholangitis. Imaging revealed the progression of hepatic metastases, biliary obstruction, and new intrahepatic cystic lesions. Blood cultures grew *Escherichia coli*, and bile cultures unexpectedly revealed motile *S. stercoralis* larvae, confirming hyperinfection. Notably, the patient had no eosinophilia. He was treated with a two-week course of ivermectin and albendazole, along with intravenous antibiotics. Given the altered gastrointestinal anatomy from prior surgery, we propose that larvae accessed the biliary system via the choledochojejunostomy. While biliary involvement was present, the migration pathway was anatomically continuous, and this presentation was considered hyperinfection rather than disseminated disease. This case underscores the importance of considering strongyloidiasis in immunocompromised patients with sepsis of unclear origin, even when eosinophilia is absent, given the high morbidity and mortality associated with hyperinfection.

## Introduction

*Strongyloides stercoralis* is a soil-transmitted nematode that can cause persistent infections in humans. While strongyloidiasis is primarily endemic to tropical and subtropical regions, cases have also been reported in temperate climates, including parts of Europe and the United States [1]. Although most cases in nontropical regions are presumed to be imported, locally acquired infections have been documented in the United States. The Centers for Disease Control and Prevention recognizes that *S. stercoralis* is endemic in certain parts of the country, with historical studies in rural Appalachia, including areas of Kentucky, Tennessee, Georgia, and West Virginia, reporting prevalence rates of approximately 1.2%-6.1% [2]. While infection is most often reported among refugees and immigrants, one study found that 59% of seropositive patients were from the United States and 70% had no documented travel to endemic areas [3], further supporting the potential for localized transmission. Although prevalence may have declined with improved sanitation, no recent national studies have been conducted, and strongyloidiasis remains a nonreportable condition in all states [2]. The World Health Organization (WHO) has historically estimated, based on data from the 1990s to early 2000s, that 30-100 million people are infected with *S. stercoralis* globally; however, the WHO acknowledges that this figure is a substantial underestimate, particularly in regions with poor sanitation and tropical climates [1].

The primary mode of transmission is through skin contact with contaminated soil, typically via walking barefoot. Uniquely, *S. stercoralis* can perpetuate infection through an autoinfection cycle in which larvae that develop within the host reinvade through the intestinal wall or perianal skin, enabling continuous reinfection without external exposure [1]. Its life cycle alternates between a parasitic phase, occurring inside the host where adult female worms lay eggs in the duodenal mucosa and jejunal lamina propria, and a free-living phase, occurring in the environment when rhabditiform larvae are excreted in stool and mature into infective forms. Rarely, other routes have been described in the literature, such as ingestion of filariform larvae via contaminated food or water or anal-oral sexual contact, but these remain uncommon and unconfirmed [4]. *S. stercoralis* can also be transmitted through solid organ transplantation, with immunosuppressed recipients, especially those on corticosteroids, at increased risk for hyperinfection or dissemination [5].

This case report presents a 56-year-old man from Jamaica with metastatic pancreatic cancer, type II diabetes mellitus, and prior Whipple procedure who developed *Escherichia coli* bacteremia from a hepatobiliary source and was ultimately diagnosed with *S. stercoralis* hyperinfection. His epidemiologic background, comorbidities, and immunocompromised state underscore the importance of considering strongyloidiasis in the differential diagnosis for sepsis in similar high-risk patients.

## Case presentation

In December 2019, a 56-year-old man, originally from Jamaica, where he had lived for 32 years before moving to the United States, with a past medical history of pancreatic adenocarcinoma status post-Whipple procedure three years prior, hypertension, and type II diabetes mellitus, and no history of human T-cell leukemia virus type 1 (HTLV-1) infection or HIV, presented for evaluation and management of liver metastases. Approximately one month earlier, he had been hospitalized elsewhere for severe abdominal pain and jaundice. Imaging at that time revealed multiple hepatic metastases and biliary obstruction, with markedly elevated bilirubin. He underwent placement of an external biliary drain and port, and liver biopsy confirmed moderately differentiated adenocarcinoma of pancreatic origin. During that admission, he developed a febrile episode managed empirically with piperacillin-tazobactam and a short course of vancomycin, though initial blood cultures were negative. He was discharged with persistently abnormal liver function tests (total bilirubin 6.8 mg/dL, aspartate aminotransferase, AST, 63 U/L, alanine aminotransferase, ALT 48 U/L, and albumin 2.3 g/dL) and referred for oncologic follow-up.

At his subsequent gastrointestinal (GI) clinic visit at Moffitt Cancer Center, he reported progressive nausea, anorexia, malaise, and discomfort around the biliary drain site. Notably, his biliary drain output had declined from approximately two bags per day to one bag per day over the preceding two weeks. In light of these symptoms, he was admitted to the hospital for further evaluation. On arrival, he was febrile (100.4°F), and his condition deteriorated rapidly, with a rising fever (102.5°F), leukocytosis (WBC 30.35 × 10³/uL), elevated procalcitonin (44.3 ng/mL), and lactic acidosis (2.8 mg/dL), consistent with severe sepsis. Admission blood cultures subsequently grew *E. coli*, and antibiotic susceptibility results are summarized in Table 1.

**Table 1:** Antibiotic susceptibility profile of Escherichia coli isolate from blood culture PDIL: phenol dilution; PINT: phenol dilution interpretation; S: susceptible; I: intermediate

Parameter	PDIL	PINT
Amikacin	≤4	S
Ampicillin/sulbactam	16	I
Cefazolin	4	I
Cefepime	≤1	S
Ceftazidime	≤2	S
Ceftriaxone	≤0.25	S
Ciprofloxacin	≤0.25	S
Gentamicin	≤1	S
Piperacillin/tazobactam	≤4	S
Tobramycin	≤1	S

**Figure 1:**
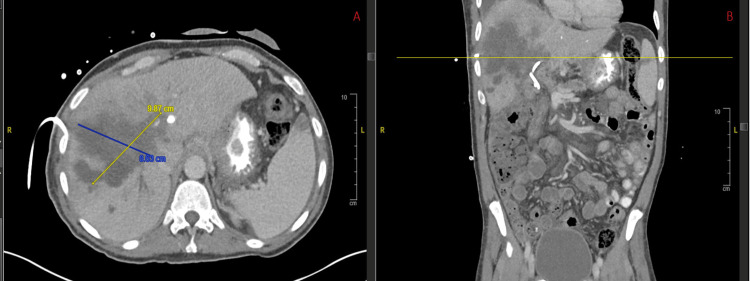
Contrast-enhanced CT scan of the abdomen. (A) Axial view showing altered perfusion of the right hepatic lobe with multiple hepatic metastases, segmental intrahepatic biliary dilation, and cystic lesions suggestive of biliary cysts or infected bilomas. (B) Coronal view demonstrating progression of hepatic metastases, near-occlusion of portal vein branches, and associated intrahepatic cystic changes

**Figure 2:**
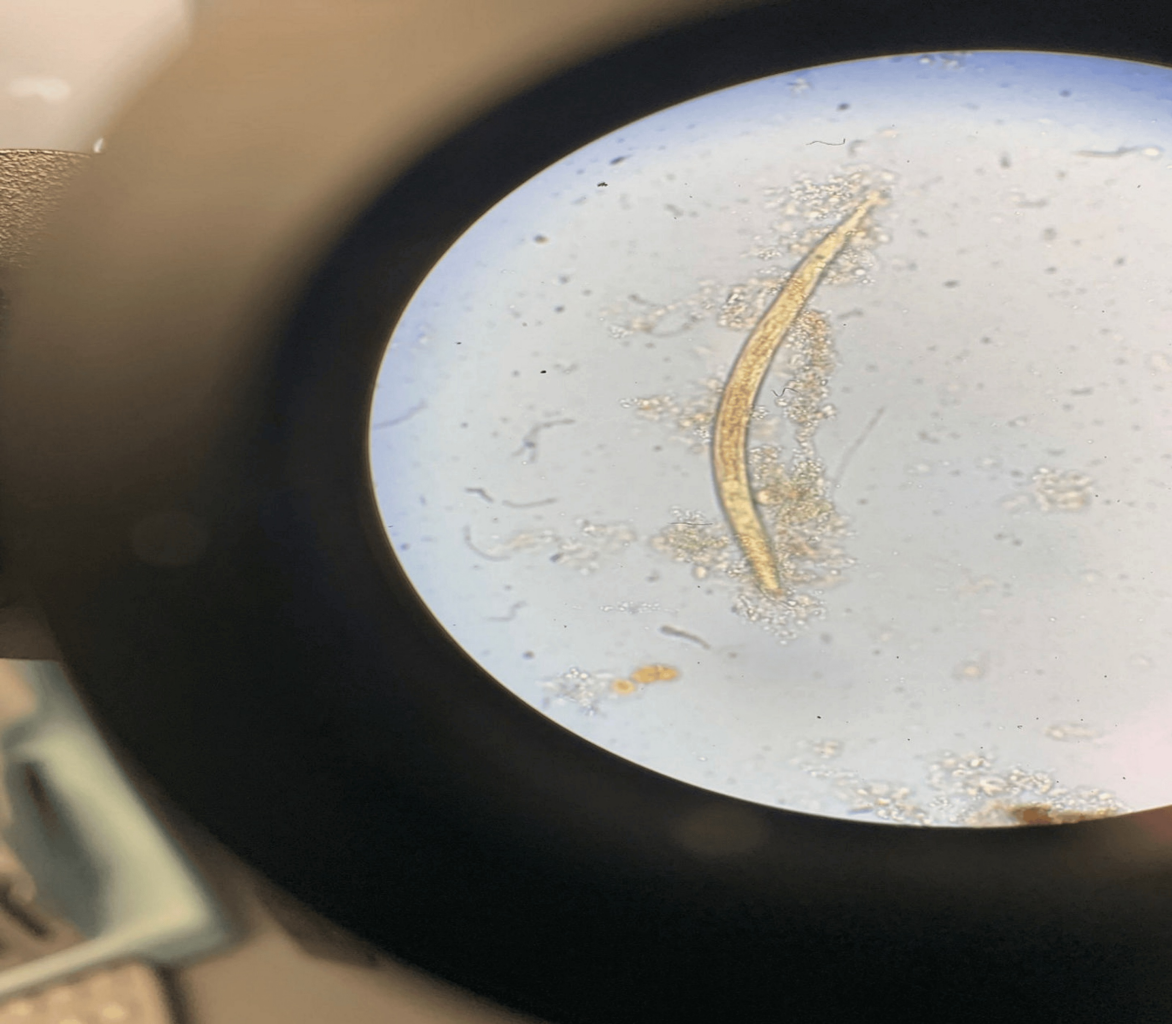
The microscopic examination of the biliary culture revealed S. stercoralis, identified by a slender, elongated nematode with tapered ends. The organism shows notable motility, characterized by a smooth and translucent body

A contrast-enhanced CT scan demonstrated altered perfusion of the right hepatic lobe, concerning for tumor vascular involvement or hepatocholangitis. There was an interval progression of hepatic metastases, increased tumor infiltration at the porta hepatis, segmental intrahepatic biliary dilation, and near-occlusion of portal vein branches. Multiple intrahepatic cystic structures suggested biliary cysts or infected bilomas (Figure 1). Liver function further deteriorated (total bilirubin 7 mg/dL, AST 569 U/L, ALT 314 U/L, ALP 262 U/L), and the patient became hypotensive (BP 88/59 mmHg). He was started on IV piperacillin-tazobactam and fluid resuscitation; planned chemotherapy was postponed due to concern for acute ascending cholangitis. Due to clinical worsening and imaging findings, his left hepatic biliary drain was exchanged. Biliary cultures were sent as part of the workup. Unexpectedly, the specimen revealed motile larvae and track marks consistent with *S. stercoralis* (Figure 2), raising concern for hyperinfection syndrome. He was promptly initiated on ivermectin 12 mg orally daily for hyperinfection. When he subsequently developed pulmonary symptoms suspected to be related to *S. stercoralis* migration, albendazole 400 mg orally twice daily was added for augmentation. Although bile cultures on December 31 were negative for *S. stercoralis*, he still completed a two-week course of combination therapy for hyperinfection. However, due to the advanced stage of his disease and overall condition, he later elected to transition to hospice care. All further treatments, including antibiotics for bacteremia, were discontinued, and he was transferred to a hospice facility two days later, where he died approximately one month after discharge.

The clinical features observed in this patient, including his immunocompromised background, biliary symptoms, and co-infection with *E. coli*, are summarized in Table 2 to highlight the rare but clinically significant presentation of biliary strongyloidiasis in the setting of altered postsurgical anatomy.

**Table 2:** Clinical features of S. stercoralis infection involving the biliary tract

Feature	Presented case
Patient background	56-year-old Jamaican man with metastatic pancreatic cancer post-Whipple
Immunosuppression	Advanced malignancy (metastatic pancreatic cancer), type II diabetes mellitus, septic shock
Route of biliary invasion	Likely via altered anatomy (choledochojejunostomy post-Whipple)
Symptoms	Fever, jaundice, nausea, anorexia, abdominal discomfort
Imaging findings	Hepatic metastases, biliary obstruction, cystic lesions (bilomas)
Laboratory findings	Elevated bilirubin, transaminitis, leukocytosis, no eosinophilia
Microbiological findings	S. stercoralis larvae identified in bile culture
Co-infection/complication	E. coli bacteremia
Treatment	Ivermectin and albendazole (completed), then hospice care
Outcome	Transitioned to comfort care after completing antiparasitic therapy

Pertinent laboratory values from the initial and follow-up hospitalizations are summarized in Table 3.

**Table 3:** Laboratory values are shown from two time points: the patient's discharge from a prior hospital admission and follow-up admission. Reference ranges are provided for clinical comparison. Marked abnormalities in liver function and sepsis markers were observed during the follow-up admission AST: aspartate aminotransferase; ALT: alanine aminotransferase; ALP: alkaline phosphatase; WBC: white blood cell

Laboratory test	Discharge from prior hospital	Follow-up admission	Reference range
Total bilirubin	6.8 mg/dL	7.0 mg/dL	0.2-1.3 mg/dL
AST	63 U/L	569 U/L	8-48 U/L
ALT	48 U/L	314 U/L	7-55 U/L
ALP	-	262 U/L	40-129 U/L
Albumin	2.3 g/dL	-	3.5-5.0 g/dL
WBC	-	30.35 × 10³/uL	4.0-11.0 × 10³/uL
Eosinophil automated count	-	0.02 k/uL	0.0-0.5 k/uL
Absolute eosinophil count	-	0.1%	1%-4%
Procalcitonin	-	44.3 ng/mL	<0.05 ng/mL
Lactic acid	-	2.8 mg/dL	0.5-2.2 mmol/L

## Discussion

This case highlights the clinical complexity and diagnostic challenges associated with *S. stercoralis* hyperinfection, particularly in immunocompromised individuals. Clinically, strongyloidiasis presents in one of four forms: acute, chronic, hyperinfection, and disseminated disease [4,6].

Acute infection typically manifests with localized pruritus at the site of skin penetration or respiratory symptoms as the larvae migrate through the lungs. Gastrointestinal symptoms such as abdominal pain and diarrhea usually develop within two weeks.

Chronic strongyloidiasis is often asymptomatic but may cause intermittent gastrointestinal complaints, such as diarrhea, vomiting, or constipation. Cutaneous findings may include larva currens, a serpiginous, rapidly migrating rash, and urticaria. Gastrointestinal hemorrhage may signal a higher parasitic burden [6].

Hyperinfection results from accelerated autoinfection and typically occurs in immunocompromised patients, including those receiving prolonged corticosteroids or those with diabetes, hematologic malignancies, or HTLV-1 infection [1,7]. Larvae proliferate rapidly, and a diagnostic hallmark includes increased larval burden in stool and sputum [6]. Enteric bacterial sepsis is a common complication, likely resulting from either translocation of gut flora by migrating larvae or inflammation-induced mucosal disruption. Without timely intervention, hyperinfection accompanied by sepsis carries a mortality rate of 87%-100% [4].

Disseminated disease occurs when larvae migrate beyond the gastrointestinal and pulmonary tracts, infiltrating organs such as the kidneys, heart, or central nervous system [6]. This stage is associated with extremely high mortality if not treated promptly.

Our patient’s case illustrates many challenges. During both his initial and follow-up hospitalizations, he exhibited leukocytosis and elevated liver enzymes but lacked eosinophilia (Eos auto 0.02 k/uL, absolute eosinophil count 0.1%). Although persistent eosinophilia is considered a hallmark of *S. stercoralis* infection, reported in up to 82% of cases [8,9], it has limited diagnostic sensitivity, especially in chronic cases, where fewer than 50% exhibit this feature [10]. This limitation is more pronounced in immunocompromised hosts, such as those with cancer or on immunosuppressive therapy [1]. The absence of hypereosinophilia in severe strongyloidiasis is thought to result from immune exhaustion due to prolonged parasitic infection and immune dysregulation associated with sepsis [6,11]. In our patient, multiple factors likely contributed, including septic shock during hospitalization, the immunosuppressive burden of metastatic pancreatic cancer, and a short course of hydrocortisone, all of which can suppress eosinophil production and recruitment. Interestingly, some data suggest that eosinophilia during hyperinfection may be associated with better outcomes, underscoring the need to interpret eosinophil counts within the broader clinical context [6].

We hypothesize that our patient likely had long-standing, unrecognized chronic strongyloidiasis, acquired earlier in life while residing in Jamaica. Supporting this, a recent seroepidemiological study reported a *S. stercoralis* seropositivity rate of 15.43% in Jamaica, with the highest prevalence among adults aged 31-50 years and significantly higher rates in rural regions [12]. His underlying immunosuppression due to metastatic pancreatic cancer likely triggered progression to hyperinfection by facilitating unchecked autoinfection and larval proliferation. Moreover, a wide array of conditions has been associated with increased risk of hyperinfection, including systemic lupus erythematosus, inflammatory bowel disease, autoimmune encephalomyelitis, tuberculosis, leukemia, malignant tumors, diabetes, congenital immunodeficiency, and HTLV-1 infection [1]. This case reinforces the importance of maintaining a high index of suspicion for hyperinfection in vulnerable populations, particularly in those with travel or residence in endemic areas, even in the absence of eosinophilia. Early recognition and prompt treatment remain essential given the significant mortality risk [4].

Diagnosis of *S. stercoralis* remains challenging. In hospital settings, stool microscopy is the most commonly used test; however, its sensitivity is limited, estimated at approximately 50% with a single sample [6,9]. Techniques such as the Kato-Katz method allow for quantification of helminth ova but are less specific for *S. stercoralis*. Serological assays, including indirect fluorescent antibody testing, gelatin particle agglutination, and polymerase chain reaction, demonstrate higher sensitivity than conventional methods [9]. Other diagnostic modalities include the string test and direct duodenal biopsy via upper endoscopy [13]. While invasive, a duodenal biopsy can provide a diagnostic yield and allow for the visualization of mucosal changes, such as ulceration, thickening of the duodenal folds, or bleeding. Nevertheless, the diagnostic gold standard remains direct visualization of larvae in stool samples or culture methods such as the Baermann technique or Koga agar plate [1,9].

The development of enteric bacteremia in the setting of *S. stercoralis* hyperinfection further complicates clinical management. It is hypothesized that migrating larvae facilitate bacterial translocation by either carrying enteric organisms through the intestinal wall or by exacerbating mucosal injury, thereby allowing bacteria to enter the systemic circulation [1]. Our patient developed *E. coli* bacteremia, a common pathogen associated with hyperinfection, and was treated promptly with intravenous piperacillin-tazobactam and fluid resuscitation for septic shock.

One of the most distinctive features of this case is the unexpected detection of *S. stercoralis* larvae in bile, a finding rarely reported in the literature. The parasite’s life cycle typically involves the skin, lungs, and gastrointestinal tract, not the hepatobiliary system. To our knowledge, only a few prior cases have reported the detection of *S. stercoralis* in bile, with a 2025 case describing it as the fourth known instance [14]. The clinical features from these previously reported cases are summarized in Table 4, demonstrating that biliary involvement may present with variable symptoms such as biliary obstruction, cholangitis, or even pancreatitis.

**Table 4:** Clinical features of S. stercoralis detected in bile from previous case reports

Study (year)	Patient presentation	Dilation of the bile duct	Larvae in stool
Delarocque Astagneau et al. [15] (1994)	Biliary obstruction	Yes	Yes
Perez-Jorge and Burdette [16] (2008)	Acute pancreatitis	No	Not mentioned
Filkins et al. [10] (2017)	Cholecystitis and extensive portal vein thrombosis	Yes	Not mentioned
Jiang et al. [14] (2025)	Gallstones and cholangitis	No	Yes

**Figure 3:**
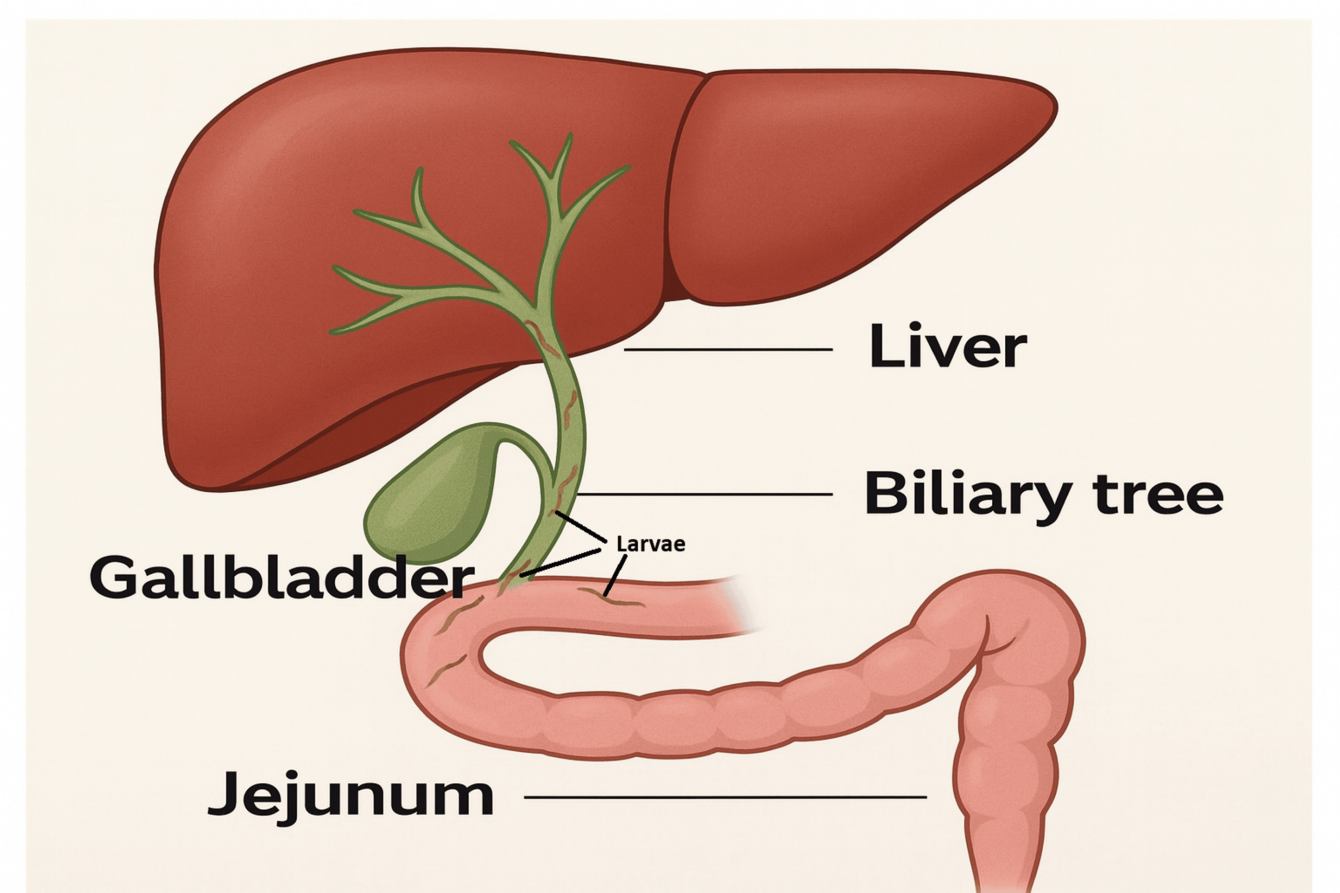
Schematic showing the proposed migration route of S. stercoralis larvae from the jejunum into the biliary tree via choledochojejunostomy following a Whipple procedure. Postsurgical anatomy creates a direct conduit between the intestinal lumen and the biliary tract, enabling parasitic entry in cases of hyperinfection Image credit: This is an original image created by the author Alan Wang

In our patient, rising liver enzymes and hyperbilirubinemia, and decreased biliary drain output preceded signs of ascending cholangitis. We suspect that a high parasitic burden contributed to cholestasis and functional biliary stasis, possibly allowing larvae to access the biliary system and introduce enteric organisms, which, in turn, resulted in *E. coli* bacteremia. The exact route of biliary invasion remains unclear. While prior studies have linked *S. stercoralis* to duodenal inflammation or papillitis, these do not fully explain the presence of larvae in bile [10,14,17,18]. A prior case involving a patient on long-term tramadol therapy hypothesized that sphincter of Oddi dysfunction allowed duodenal content reflux, permitting larvae to enter the biliary tree [10]. However, our patient had no history of long-term opioid use. Instead, we propose that chronic strongyloidiasis, likely acquired in early life, combined with his post-Whipple anatomy, in which the common bile duct is anastomosed directly to the small intestine, created an open conduit for intestinal larvae to migrate into the biliary tree. This hypothesis is supported by prior studies reporting that approximately 70% of patients undergoing hepatobiliary resection with choledochojejunostomy had positive bile cultures, and that cholangitis from reflux of intestinal contents across the anastomosis occurred in about 10% of cases [19]. Experimental animal studies have also confirmed the occurrence of intestinal-biliary reflux after anastomosis of the common bile duct to either the duodenum or jejunum [20]. These findings reinforce the plausibility that altered postsurgical anatomy can facilitate retrograde migration of enteric organisms, including *S. stercoralis* larvae, into the biliary system. A schematic illustration of this proposed route is shown in Figure 3. Although detection of larvae in bile is unusual, we considered this presentation to represent hyperinfection rather than disseminated strongyloidiasis. In line with clinical descriptions, disseminated disease is generally considered when larvae are found in organs beyond the gastrointestinal and pulmonary systems, such as the central nervous system, where they may occasionally be detected in cerebrospinal fluid. In our patient, the biliary involvement was plausibly explained by retrograde migration through an anatomically continuous pathway created by the choledochojejunostomy. This pattern is more consistent with intensified autoinfection in the setting of immunosuppression and altered surgical anatomy.

Management of *S. stercoralis* infection is primarily pharmacologic, but in the setting of hyperinfection or disseminated disease, particularly in critically ill patients, a comprehensive, multidisciplinary approach is essential. Ivermectin is the treatment of choice, offering high efficacy in clearing larvae with a more favorable side effect profile than older agents such as thiabendazole, which has fallen out of favor due to frequent and unpleasant adverse effects [1,6]. The standard ivermectin regimen is 200 μg/kg/day for two consecutive days, though extended courses, up to two weeks or until clinical improvement and negative stool or sputum microscopy, are often recommended in hyperinfection [1,4]. Mebendazole has limited utility due to poor absorption, while albendazole may be used as a secondary agent but carries a risk of pancytopenia [6]. In critically ill patients, antiparasitic therapy should be integrated with general supportive measures, including hemodynamic stabilization with fluids and vasopressors for septic shock, respiratory support when pulmonary involvement is present, and early broad-spectrum antibiotics to address secondary bacterial translocation. Infection control measures are important to prevent nosocomial spread, and nutritional optimization, such as enteral feeding, can support immune recovery. Follow-up stool microscopy two to four weeks after treatment completion is recommended to confirm parasite clearance.

A notable limitation of this case report is the inability to assess the long-term clinical impact of antiparasitic therapy. Although the patient’s bile cultures were negative for *S. stercoralis* prior to discharge and he completed a full two-week course of ivermectin and albendazole, his advanced metastatic pancreatic cancer prompted a transition to hospice care shortly after treatment completion. Consequently, while parasitological clearance was documented in the short term, there was no opportunity to evaluate sustained eradication, resolution of hyperinfection, or survival benefit. This limits the ability to draw conclusions regarding the long-term efficacy of early intervention in similar high-risk patients.

## Conclusions

This case describes an unusual presentation of *S. stercoralis* hyperinfection in which post-Whipple anatomy likely enabled biliary migration of larvae without true dissemination. The absence of eosinophilia, despite heavy parasitic burden and septic shock, highlights the limited sensitivity of this finding in immunocompromised patients. Clinicians should consider strongyloidiasis in at-risk individuals, particularly those with altered gastrointestinal anatomy and epidemiologic exposure, presenting with sepsis of unclear origin. Multidisciplinary management is essential in critically ill cases. Although the patient achieved parasitological clearance before discharge, transition to hospice shortly thereafter precluded assessment of long-term outcomes.

## References

[1] Yang R, Xu M, Zhang L, Liao Y, Liu Y, Deng X, Wang L (2025). Human Strongyloides stercoralis infection. J Microbiol Immunol Infect.

[2] Centers for Disease Control and Prevention (2013). Notes from the field: Strongyloidiasis in a rural setting---Southeastern Kentucky, 2013. MMWR Morb Mortal Wkly Rep.

[3] Kottkamp A, Mehta S (2018). 1137. Implementation of universal screening for Strongyloidiasis among solid-organ and hematopoietic stem cell transplantation candidates in a non-endemic area. Open Forum Infect Dis.

[4] Czeresnia JM, Weiss LM (2022). Strongyloides stercoralis. Lung.

[5] Ofosu A, Higgins J, Frye JS, Kumari R, Barakat MT (2021). Strongyloides superinfection after liver transplantion. Dig Dis Sci.

[6] Keiser PB, Nutman TB (2004). Strongyloides stercoralis in the immunocompromised population. Clin Microbiol Rev.

[7] van Tong H, Brindley PJ, Meyer CG, Velavan TP (2017). Parasite infection, carcinogenesis and human malignancy. EBioMedicine.

[8] van Balkum M, Kluin-Nelemans H, van Hellemond JJ, van Genderen PJJ, Wismans PJ (2018). Hypereosinophilia: a diagnostic challenge. Neth J Med.

[9] Khurana S, Sethi S (2017). Laboratory diagnosis of soil transmitted helminthiasis. Trop Parasitol.

[10] Filkins LM, Gaston DC, Mathison B (2017). Biliary Strongyloides stercoralis with cholecystitis and extensive portal vein thrombosis. Open Forum Infect Dis.

[11] Lin Y, Rong J, Zhang Z (2021). Silent existence of eosinopenia in sepsis: a systematic review and meta-analysis. BMC Infect Dis.

[12] Chin AV, Thompson T, Denton CS, Lindo JF (2024). The seroepidemiology of Strongyloides stercoralis infection in Jamaica. J Trop Med.

[13] Siddiqui AA, Berk SL (2001). Diagnosis of Strongyloides stercoralis infection. Clin Infect Dis.

[14] Jiang XH, Deng Q, Wu ZK, Li JZ (2025). Alive Strongyloides stercoralis in biliary fluid in patient: a case report. World J Gastroenterol.

[15] Delarocque Astagneau E, Hadengue A, Degott C, Vilgrain V, Erlinger S, Benhamou JP (1994). Biliary obstruction resulting from Strongyloides stercoralis infection. Report of a case. Gut.

[16] Perez-Jorge EV, Burdette SD (2008). Association between acute pancreatitis and Strongyloides stercoralis. South Med J.

[17] Ikeuchi N, Itoi T, Tonozuka R (2016). Strongyloides stercoralis infection causing obstructive jaundice and refractory pancreatitis: a lesson learned from a case study. Intern Med.

[18] Hirata T, Kishimoto K, Kinjo N, Hokama A, Kinjo F, Fujita J (2007). Association between Strongyloides stercoralis infection and biliary tract cancer. Parasitol Res.

[19] Sugawara G, Ebata T, Yokoyama Y, Igami T, Takahashi Y, Takara D, Nagino M (2013). The effect of preoperative biliary drainage on infectious complications after hepatobiliary resection with cholangiojejunostomy. Surgery.

[20] Musgrove JE, Grindlay JH, Karlson AG (1952). Intestinal-biliary reflux after anastomosis of common duct to duodenum or jejunum: an experimental study. AMA Arch Surg.

